# Phylogenetic Analysis and Spread of HPAI H5N1 in Middle Eastern Countries Based on Hemagglutinin and Neuraminidase Gene Sequences

**DOI:** 10.3390/v17050734

**Published:** 2025-05-20

**Authors:** Laith N. AL-Eitan, Diana L. Almahdawi, Iliya Y. Khair

**Affiliations:** Department of Biotechnology and Genetic Engineering, Jordan University of Science and Technology, Irbid 22110, Jordan; dlalmahdawi20@sci.just.edu.jo (D.L.A.); iykhair19@sci.just.edu.jo (I.Y.K.)

**Keywords:** avian influenza, H5N1, phylogenetic analysis, biosafety, biosecurity, surveillance

## Abstract

Highly pathogenic avian influenza (HPAI) A/H5N1 viruses threaten animal and human health worldwide. The first documented cases in the Middle East were reported in 2005; however, despite extensive phylogenetic studies, there is limited information on the transmission dynamics of the virus within this region. We analyzed HA and NA gene sequences from various hosts to address this gap and to understand the virus’s spread and evolution in the Middle East. We hypothesized that H5N1 transmission exhibits host-specific or geographically influenced clade structures in this region. This study traced transmission pathways of HPAI A/H5N1 through a phylogenetic and amino acid sequence analysis of HA and NA gene segments from isolates across different hosts in Middle Eastern countries, using the MUSCLE algorithm for alignments and MEGA11 software for phylogenetic analysis. Sequences were selected from NCBI’s virus database based on geographic and host diversity, including those from birds, humans, and other mammals, and were collected at different time points, predominantly after the early 2000s. An amino acid phylogenetic tree was also constructed to examine the conservation of key HA and NA protein residues, identifying distinct clades linked to specific countries and host species, suggesting a possible interspecies transmission and cross-border spread distinct between Egypt and neighboring countries. These findings underscore the role of migratory birds in regional transmission and point to the need for more targeted surveillance and biosecurity efforts, offering more genomic insights into the spread of HPAI A/H5N1 and contributing valuable information for future prevention strategies.

## 1. Introduction

Since their emergence, avian influenza viruses have posed a significant global risk to public health due to their extensive spread and substantial mortality rates [1]. Among them, the H5N1 subtype of highly pathogenic avian influenza (HPAI) is of particular concern due to its zoonotic potential and severe impact on animal and human health. Although it mainly affects birds, certain strains have developed the ability to cross over to humans, causing severe respiratory illnesses that often require intensive care [2]. As defined by the World Health Organization, avian influenza is an infectious disease caused by a rapidly evolving group of subtypes that affect a wide range of species, including birds, poultry, and humans [3]. The influenza A (H5N1) virus, species Alphainfluenzavirus influenza, belongs to the family Orthomyxoviridae, having two antigens on the glycoprotein surface—hemagglutinin (HA) and neuraminidase (NA)—which are critical for the virus’s entry into host cells and its pathogenicity. Recently, 18 HA and 11 NA subtypes have been circulating and identified globally [2,4]. Small changes in these proteins can lead to antigenic variation, particularly when a single mutation occurs at a key site on the HA protein. This may make the virus antigenically different [5]. HA plays a central role in vaccine design and antigenic drift, making it essential to global surveillance systems like the WHO’s Global Influenza Surveillance and Response System (GISRS); changes in the HA protein often trigger the need for vaccine updates, while tracking its variants offers direct insight into strain evolution, immune escape, and regional outbreak preparedness [5,6,7]. Similarly, NA is responsible for viral release and propagation; it also influences viral transmissibility and pathogenicity, especially through mutations affecting antiviral drug susceptibility, particularly neuraminidase inhibitors such as oseltamivir [8]. The World Health Organization (WHO), the World Organization for Animal Health (OIE), and the Food and Agriculture Organization (FAO) established a classification system for H5N1 into diverse clades and subclades based on the hemagglutinin (HA) gene sequence, which helps track the genetic evolution and geographic spread of the virus [9]. Each clade represents viral groups that all share a common ancestor and meet defined nucleotide divergence thresholds, typically 1.5% or more in the HA gene [10]. Subclades are further subdivided, reflecting additional evolutionary divergence; for example, subclade 2.2.1 descended from clade 2.2 and was linked to extensive outbreaks in Egypt from 2006 to 2010, infecting poultry and humans [11]. Incorporating clade and subclade data into phylogenetic analysis enhances the ability to detect host specificity, track geographical spread, and interpret temporal trends, which is vital for guiding surveillance strategies and biosecurity policies in affected regions [9,10].

Wild birds, particularly those in the Charadriiformes and Anseriformes orders, are recognized as the primary transmission route, where infected birds release a significant amount of the virus through their feces and respiratory tract; these avian influenza strains circulate in two forms: Low-Pathogenicity avian influenza (LPAI) and High-Pathogenicity avian influenza (HPAI). Although LPAI typically causes mild or no symptoms, HPAI—especially the H5N1 subtype—can cause severe disease and high mortality in birds and occasionally lead to serious illness in humans. However, human cases remain rare [12,13]. In 1996, the first H5N1 HPAI virus was found in sick geese in Guangdong, China, where its zoonotic potential became evident with the first human infections [14]. The Middle East had its first H5N1 outbreak between 2005 and 2006, initially affecting poultry in countries such as Turkey, Iraq, and Egypt, quickly spreading to other nations and infecting humans [15,16,17,18]. Turkey became the first Middle Eastern country to report human cases of H5N1 in January 2006, when several individuals, primarily children, developed severe respiratory symptoms after contact with infected poultry [19]. Shortly after Iraq reported its first human case of H5N1 in January 2006, a teenage girl in northern Iraq succumbed to the virus, marking the first fatality in the country [18]. In February 2006, H5N1 was first detected in poultry in Egypt, with human cases following soon after. By 2008, Egypt had the highest number of confirmed human H5N1 infections outside of Asia, primarily attributed to the proximity between humans and domestic poultry in rural areas, where backyard farming is common, and biosecurity measures were difficult to enforce [20,21]. Note that H5N1 poses a significant risk to human and animal health, with a fatality rate of approximately 60% due to its ability to cause severe respiratory complications [22,23,24]. The virus spreads through direct contact, contaminated environments, and respiratory droplets, making it difficult to contain once an outbreak occurs [25]. Mutations in the HA1 and HA2 genes of the H5N1 subtype increase its pathogenicity by altering the HA0 connecting peptide. This modification allows trypsin-like proteases to cleave HA0 in several body systems beyond the respiratory tract, leading to extensive viral replication, rapid spread, and high mortality [26]. Despite ongoing surveillance, there is still limited information about H5N1 transmission and evolution in the Middle East. While global studies exist, few focus on the virus’s genetic diversity and epidemiology in this region, which makes it harder to develop effective prevention strategies in areas with recurring outbreaks. This study aims to fill gaps by focusing on the hemagglutinin (HA) and neuraminidase (NA) genes for phylogenetic and evolutionary analysis, given their critical roles in viral entry, release, host specificity, immune recognition, and antiviral response; HA remains the main target of neutralizing antibodies, while NA mutations are key to understanding viral adaptation, antigenic drift, and antiviral resistance, making both genes relevant for studying viral evolution and vaccine design [27,28]. We hypothesize that host species and geographic factors shape distinct clades and transmission patterns in H5N1, looking at differences in HA and NA evolution across regions and hosts. Understanding these patterns will offer critical insights into how specific hosts and environments contribute to viral spread, antigenic drift, and resistance development, ultimately guiding more targeted surveillance, vaccine updates, and outbreak control strategies.

## 2. Methodology

### 2.1. Study Design and Inclusion/Exclusion Criteria

#### 2.1.1. Nucleotide Sequences

This study included viral sequences from Middle Eastern countries (Iran, Iraq, Israel, Kuwait, Lebanon, Turkey, Saudi Arabia, and Egypt) found in the NCBI Virus database. To ensure data quality and meaningful comparative analysis, we permitted a 40-base-pair variation in nucleotide count, with a global reference length of 1760 base pairs; we selected sequences between 1720 and 2000 base pairs in length. This balanced the need to accommodate natural length variations and minimizing the risk of including anomalous sequences. Host-wise, we include sequences from humans, chickens, and turkeys, given the commonly observed and significant impact of the virus on these species in the region. After the application of the initial length- and host-based filters, 211 sequences remained from 2730. An additional filtration step was performed by utilizing NCBI BLAST version 2.16.0 to exclude sequences with 100–99% identity and remove duplicates, with particular attention given to differences in query coverage and base-pair length. This ensured the elimination of redundant sequences, thereby reducing potential biases and enhancing the diversity of the dataset. The collection date of each sequence was prioritized over the release date to ensure biological relevance in the phylogenetic analysis, particularly for studies of evolutionary patterns and transmission dynamics. In cases where sequences shared identical parameters, the most recently submitted data were selected to use the most up-to-date information. As part of the final filtering step, we removed sequences with unresolved regions, ambiguous nucleotides, or middle gaps to avoid inaccuracies caused by poor sequencing quality; these steps resulted in a final dataset of 62 high-quality sequences for analysis, ensuring a robust foundation for the study’s evolutionary and phylogenetic assessments.

#### 2.1.2. Amino Acid Sequences

The same filtration method was used for protein collection. Different years and months and hosts (humans, chickens, turkeys) from the previously mentioned Middle Eastern countries were considered. We selected HA and NA amino acid sequences with a total size of 568 and 449, respectively, allowing up to 4 amino acid differences to maintain sequence diversity without introducing excessive variability. Duplicates and repetitive sequences were avoided, and any ambiguous sequences were removed. All chosen data were carefully aligned to ensure quality and comparability across strains.

### 2.2. Molecular Evolutionary Genetics Analysis (MEGA) Version 11

MEGA11 software was chosen for this study due to its well-established performance in handling large datasets and complex evolutionary models [29]. Moreover, MAFFTv7 was initially used to ensure accuracy in nucleotide alignment due to its high sensitivity and fast performance when working with multiple sequences [30]. Following the primary alignment, MUSCLE was used in MEGA 11 to refine and validate the alignment, ensuring that no significant regions were misaligned, utilizing the default gap penalties (Gap Open value of −400.00 and Gap Extend value of 0.00) to preserve balance between gap creation and alignment accuracy, reducing excessive insertions or deletions that might distort evolutionary relationships [31]. The Neighbor-Joining (NJ) method was selected for phylogenetic tree construction due to its computational efficiency and reliable accuracy in inferring evolutionary relationships from sequence data, making it suitable for analyzing large datasets in a reasonable timeframe [32]. However, we chose the p-distance model to ensure direct and straightforward evolutionary distance measurements that calculate the proportion of nucleotide differences between sequences [33]. We also applied both a Gamma distribution to account for evolutionary rate differences across sites, which is critical given that different parts of the viral genome may evolve at different rates, and the Nearest-Neighbor-Interchange (NNI) algorithm to optimize tree topologies efficiently while keeping computational demands low [34,35]. Finally, a complete deletion was employed for gaps and missing data, ensuring that only fully resolved and reliable sequence regions were included in the analysis, reducing potential bias from incomplete data. A bootstrap analysis with 1000 replicates was conducted for NJ trees to provide statistical support for the inferred phylogenetic relationships, ensuring that the tree structures were reliable [36]. Lastly, we used the outgroup method to root the phylogenetic trees, which helped in tracing divergence patterns, understanding how more recent strains evolved, and clarifying lineage relationships supporting the temporal and geographic clustering observed across the dataset [37].

### 2.3. Phylogenetic Analyses

The tree-building process using the Neighbor-Joining (NJ) method was repeated at least 10 times to verify the consistency and stability of the resulting phylogenies. This repetition was necessary to ensure that minor computational variations did not affect the overall results, providing a high degree of confidence in the stability of the tree topology. As mentioned, removing similar sequences was critical to avoid redundancy and ensure accurate phylogenetic representation. Note that sequences with high similarity (often clones or variations of the same strain) were filtered out as their presence can artificially skew the tree into a “ladder-like” structure, reducing the precision and confidence (bootstrap values) of phylogenetic relationships. Of course, this issue was mitigated by applying the rigorous filtering process described earlier, leaving only the distinct, representative sequences. Likewise, the protein phylogenetic tree was constructed using the Neighbor-Joining (NJ) method with bootstrap 1000; each tree was made separately with its unique available data using the outgroup rooting method; and the protein IDs for these sequences were sourced from NCBI too, ensuring consistency in the data used for both nucleotide- and protein-based analyses. Bootstrap values below 50% were excluded from the visualized tree to enhance clarity in the final results. This allows the focus to remain on branches with statistically solid support and presents a more reliable view of evolutionary relationships. However, it was observed that conserved regions were primarily located at the beginning and end of the sequences, while the central areas exhibited more variability. This finding was systematically documented by carefully recording all variable sites for the NA and HA amino acid changes, which were then compiled into tables for further comparative analysis. After generating the base topology using the Neighbor-Joining (NJ) method, we constructed a molecular clock tree using the same amino acid trees to estimate divergence timing and observe potential temporal clustering. Using the RelTime method in MEGA11, we calibrated the tree by assigning sample collection years as tip dates, allowing the software to estimate divergence times relative to these known time points [29,38].

### 2.4. Amino Acid Analysis

The amino acid analysis was carried out manually after the sequence alignment using MEGA 11 software, as detailed in Supplementary Tables S1 and S2. The sequences were selected based on their geographical relevance, evolutionary importance, and representation across different hosts and timeframes. The global reference sequence (Goose Guangdong 1996), the oldest and first reported strain, was included as a baseline, followed by regional reference strains, such as ACA21577.1 Chicken Kuwait 2007 and AEX88483.1 Turkey Turkey 2008 for HA protein, and AOE23190.1 Chicken Lebanon 2016 and ABQ58915.1 Turkey Turkey 2005 for NA protein, which were chosen to represent key Middle Eastern strains. A single representative sequence from each country was selected for HA (AQS24398.1 Chicken Egypt 2016, AMN14712.1 Chicken Egypt 2015, ALJ33536.1 Turkey Israel 2015, AKC44576.1 Human Egypt 2014, AEP37319.1 Chicken Egypt 2011, AEN68619.1 Turkey Israel 2011, CBW54817.1 Chicken Egypt 2010, ADI58758.1 Chicken Israel 2010, ADI58758.1 Chicken Israel 2010, AFW18183.1 Chicken Menofia 2009, AFW18314.1 Chicken Gaza 2008). However, since the NA sequence data pool was smaller, all available sequences were included in the comparison (ACJ53876.1 Human Egypt 2008, ABQ58919.1 Human Turkey 2006, ABV24001.1 Human Iraq 2006, ACJ53845.1 Human Egypt 2006, ACJ53847.1 Human Egypt 2007, ACR56188.1 Chicken Egypt 2007, ACT15358.1 Human Egypt 2009, ADF31753.1 Chicken Egypt 2008, ADG21450.1 Human Egypt 2010, ADI23974.1 Turkey Egypt 2009, AFW18449.1 Chicken Menofia 2010, AHI43550.1 Chicken Egypt 2012, AIO11742.1 Human Egypt 2014, AIX94868.1 Chicken Egypt 2013, AJM70748.1 Human Egypt 2014, AKP04579.1 Chicken Israel 2015, AKP04583.1 Turkey Israel 2015, AKP04584.1 Chicken Qalqilya 2015, AKP04585.1 Turkey Jenin 2015, ANJ61441.1 Human Egypt 2014, AQS24383.1 Chicken Egypt 2016, AQS24389.1 Chicken Egypt 2016, AQS24409.1 Chicken Egypt 2016, CBW54822.1 Chicken Egypt 2009), with those having close IDs and dates removed to avoid redundancy.

These sequences span from 2007 to 2016 and cover various hosts, with the criteria for inclusion based on date, country of origin, and host species, ensuring that the dataset was diverse enough to provide meaningful insights into mutation and evolution patterns as previously addressed in the section outlining the inclusion and exclusion criteria, mainly focusing on the impact of bird migration in viral transmission. The primary objective of the amino acid alignment was to identify specific site changes and assess their potential functional impacts; the alignment dots in both Supplementary Tables S1 and S2 represent amino acids that were identical to the global reference sequence (Goose Guangdong 1996), while uncolored columns highlight conserved regions across all organisms typically associated with essential viral functions. Yellow-highlighted columns denote variable regions, further classified into parsimony-informative sites (Pi) and singleton sites (S), indicating the evolutionary significance of these changes. Only amino acid regions recurrent in more than three sequences were included to minimize errors from personal or sequencing machine inaccuracies, reducing the likelihood of error and ensuring that the observed changes reflected genuine evolutionary mutations. Repetitive sequence changes identified across multiple sequences suggested actual evolutionary events in these regions (Supplementary Table S2). An asterisk (*) indicates a low-frequency change that is still considered significant. In contrast, a double asterisk (**) indicates high-frequency changes across multiple sequences, which are particularly relevant for understanding viral adaptation and evolution.

## 3. Results

### 3.1. Phylogenetic Tree Analysis

**Figure 1:**
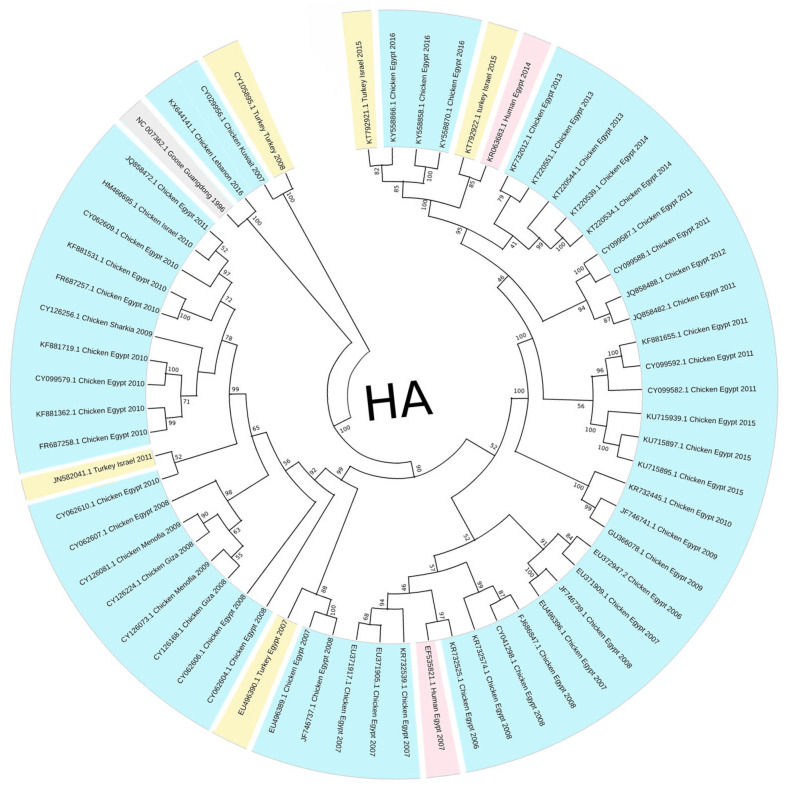
This figure demonstrates the 50% confidence cut-off for the 62 collected HA sequences from H5N1 viruses isolated from different hosts in Middle Eastern countries. All sequences in this phylogenetic tree were downloaded from the NCBI Virus database. The results show the relationship between the chosen sequences and that with the original goose-isolated H5N1 strain found in Guangdong, China (reference sequence). The primary basis for the color scheme is host species, with blue representing chicken isolates, yellow indicating turkey isolates, pink representing human isolates, and gray indicating the reference or outgroup strain.

**Figure 2:**
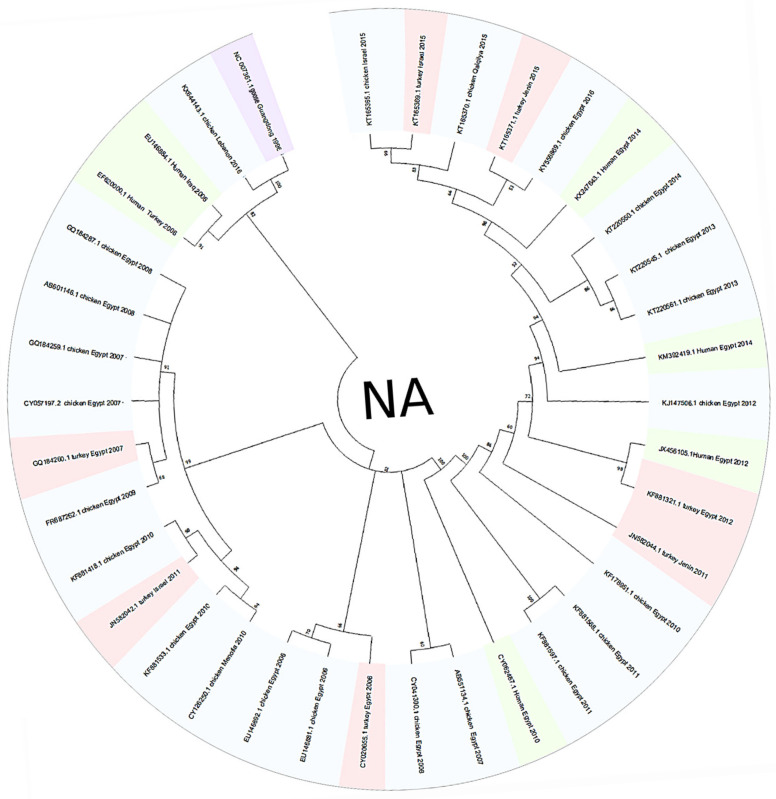
This figure demonstrates the 50% confidence cut-off for the 32 collected NA sequences from H5N1 viruses isolated from different hosts in Middle Eastern countries. All sequences used in this phylogenetic tree were downloaded from the NCBI Virus database. The results show the relationship between the selected sequences, with the original goose-isolated H5N1 strain from Guangdong, China, included as a reference sequence. The color scheme in the phylogenetic tree is based on host species, where blue represents chicken isolates, green indicates human isolates, pink represents turkey isolates, and purple indicates the reference or outgroup strain.

The HA phylogenetic tree structure, including 62 sequences from H5N1 isolates across the Middle East (Figure 1), shows significant evolutionary relationships between the selected sequences and the reference strain, since the first H5N1 strain was initially isolated from a goose in Guangdong, China. Additionally, tight clustering is seen in most Egyptian isolates (primarily chicken-derived from 2007 to 2015), forming several well-supported subclades and highlighting continued viral circulation. The outgroup rooting method using Goose/Guangdong 1996 as a reference strain anchors the tree since it is the most basal lineage; this confirms the ancestral position relative to all included Middle Eastern strains. Other unique nodes include isolates from Gaza, Menofia, and Kuwait, each forming smaller subclades or pairing with sequences from neighboring regions. Furthermore, lengths were also variable, with shorter branches between closely related Egyptian strains and longer ones connecting geographically distant or temporally earlier samples. However, the NA gene phylogenetic analysis (Figure 2) also revealed a structured clustering pattern consistent with geographic origin and host species, with moderate-to-high bootstrap values across major nodes supporting the reliability of the clustering. This tree shows a clearer relationship between human and chicken virus transmission, with specific clades clustering closely together, indicating that Egypt dominated most sequences. Older strains like Turkey 2005 and early human isolates showed greater divergence, while more recent isolates from Egypt (2015–2016) demonstrated tighter clustering with minimal branch lengths.

### 3.2. Amino Acid Analysis

**Figure 3:**
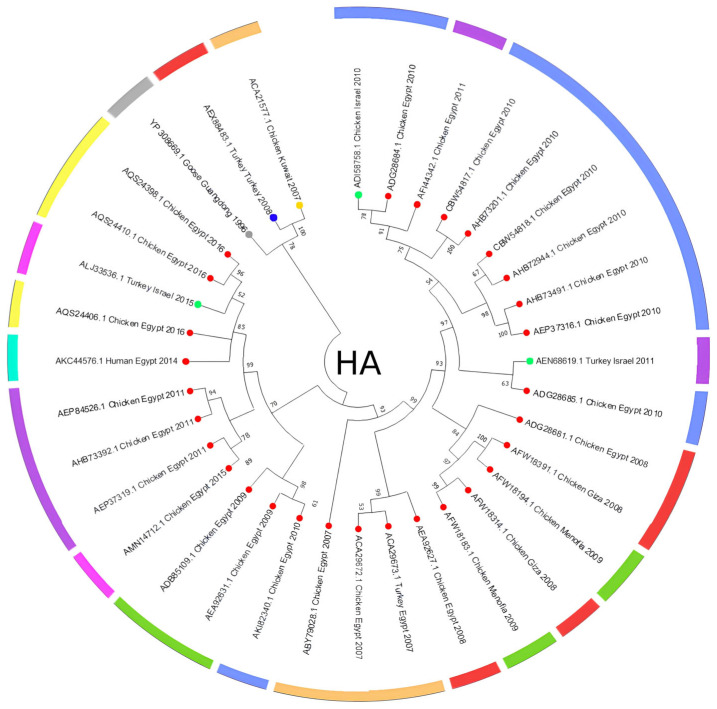
After the application of the tree cut-off, the amino acid tree contains 62 aligned HA protein sequences with a confidence value of over 50%. All sequences in this phylogenetic tree were downloaded from the NCBI Virus database. HA protein relatedness was discovered at a significant level among H5N1 strains that spread throughout the Middle East compared to the original primary sequence from Guangdong, China (reference sequence). The phylogenetic tree uses colored branch tip circles to indicate the countries of origin for the isolates: red for Egypt, green for Israel, blue for Turkey, and gray for the reference strain from China. The colored segments in the outer ring represent the year of isolation: gray for the oldest reference strain from 1996, orange for 2007, red for 2008, green for 2009, blue for 2010, purple for 2011, light green for 2014, pink for 2015, and yellow for 2016.

**Figure 4:**
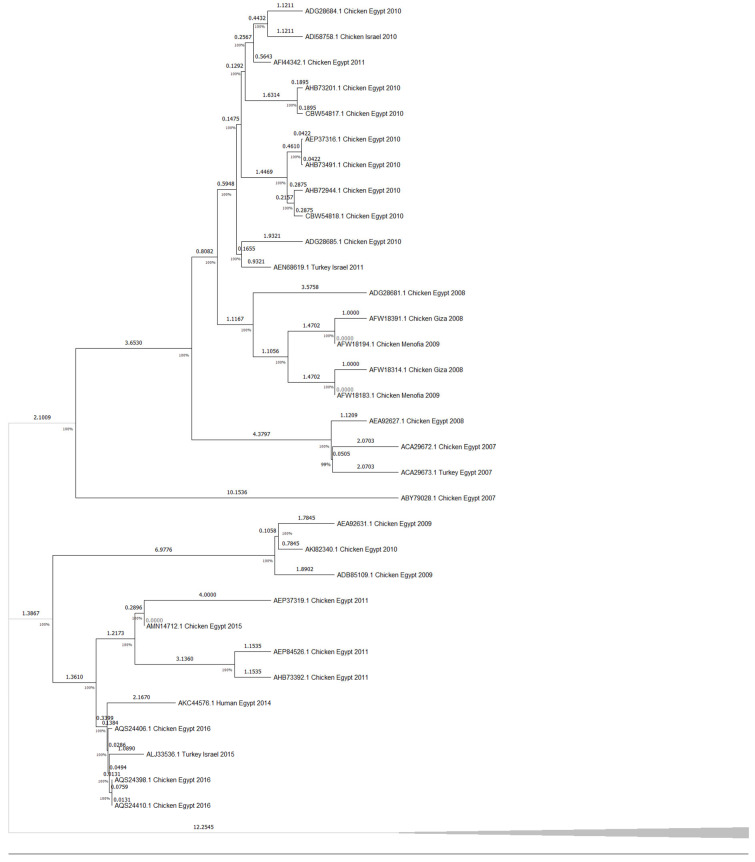
Time-calibrated phylogenetic tree of H5N1 HA protein sequences inferred using the RelTime method in MEGA11, using 35 HA protein sequences from H5N1 isolates collected between 2006 and 2016 in Egypt, Israel, Turkey, and Gaza. Branch lengths represent relative divergence times, and values at internal nodes indicate the estimated time from the most recent common ancestor, where short branch lengths indicate high genetic similarity and fewer mutations between sequences. The tree highlights the temporal clustering of sequences by year and geography, with older isolates branching near the base and more recent sequences forming distal clades.

**Figure 5:**
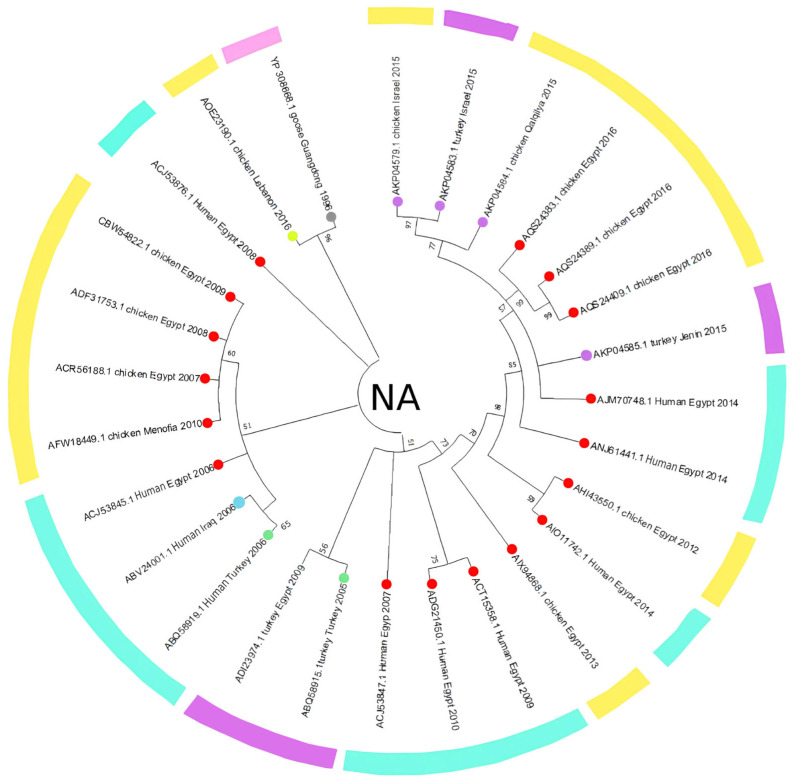
The phylogenetic tree of NA amino acid sequences from H5N1 viruses isolated in Middle Eastern countries, downloaded from the NCBI Virus database and analyzed using MEGA11. The tree shows clustering based on geographic origin and year of isolation, with Egyptian strains dominating the recent clusters and older isolates from Turkey and Iraq forming more distinct branches. Bootstrap values (≥50%) are displayed at major nodes. The phylogenetic tree uses colored branch tip circles to indicate the countries of origin for the isolates: red for Egypt, purple for Israel, light green for Lebanon, light blue for Iraq, green for Turkey, and gray for China (reference strain). The colored segments in the outer ring represent the host species: gray for goose (reference strain), yellow for chicken, light green for human, and purple for turkey.

**Figure 6:**
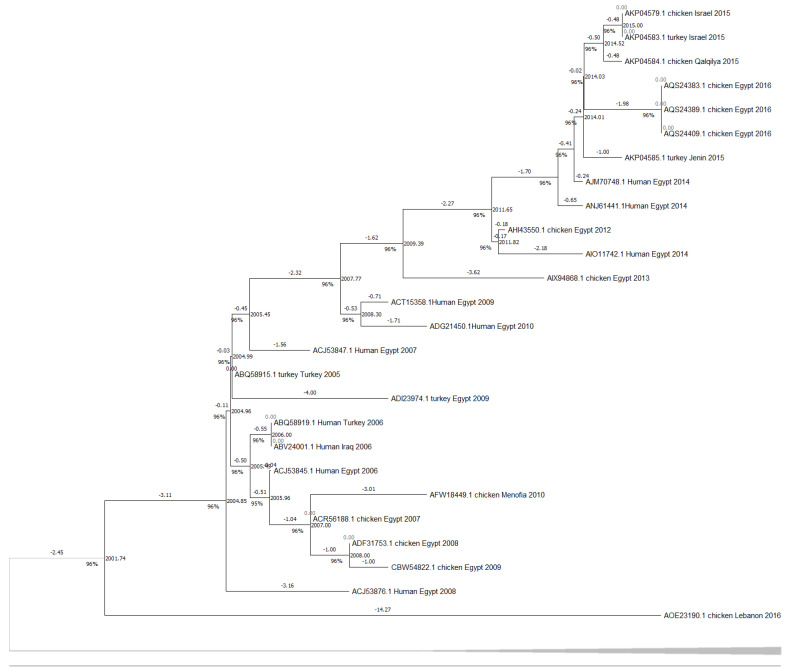
This time-calibrated phylogenetic tree of H5N1 NA protein sequences was created using the RelTime method in MEGA11, using 35 HA protein sequences from H5N1. Divergence times are indicated along branches, with bootstrap values ≥50% shown at nodes. The tree highlights evolutionary relationships over time, with older strains like Turkey 2005 showing greater divergence, while more recent Egyptian isolates cluster tightly with shorter branch lengths. Sequences were sourced from the NCBI Virus database and analyzed using MEGA11 under a strict molecular clock model.

The HA amino acid-based phylogenetic tree, including 35 amino acid sequences from H5N1 isolates across the Middle East (Figure 3), shows an apparent clustering of sequences by both geographic origin and collection year, with Egyptian isolates forming the majority of the tree and showing multiple subclusters, while sequences from Turkey, Israel, and Kuwait are grouped separately. However, to estimate divergence timing and observe potential temporal clustering, we constructed a molecular clock tree using the HA protein dataset in MEGA11 (Figure 4); the analysis also included 35 amino acid sequences across isolates from Egypt, Israel, Kuwait, and Turkey, representing a curated subset of the broader dataset for which accurate collection years were available. Notably, several internal branches have bootstrap values above 100%, which confirms the statistical reliability of the grouping and branching order. Interestingly, several recent isolates, such as AQS24410.1, AQS24398.1, and AQS24406.1 (Chicken Egypt 2016), are clustered closer to the root despite their more recent collection dates, showing minimal genetic distance values (as low as 0.0131 and 0.0286). In contrast, earlier isolates like ACA29672.1 (Chicken Egypt 2007) and ABY70828.1 (Chicken Egypt 2007) are positioned further from the root with higher divergence values (up to ~2.8793), indicating greater evolutionary distance from the ancestral node and supporting a pattern of distinct sub-lineage development over time. Short branch lengths (e.g., values close to 0.0000) in phylogenetic trees indicate high genetic similarity and fewer mutations between sequences, suggesting a more recent common ancestor and stronger evolutionary relatedness [38,39]. This clock model tree highlights how regional strains evolved in parallel, with some showing longer branches, suggesting more rapid divergence or local adaptation. Similarly, the NA amino acid-based phylogenetic tree, which includes 32 amino acid sequences (Figure 5), supports sequences other than those identified in Figure 3. Although the Egyptian isolates form most of the tree and display multiple subclusters, sequences from Turkey, Israel, and Lebanon are still grouped separately with modest branch support. This tree shows how the Lebanon 2016 isolate clusters closely with the reference sequence alongside several human and animal strains that are grouped tightly together. A molecular clock was also made using the same NA amino acid dataset in MEGA11 (Figure 6). Notably, there is clear evolutionary divergence over time. Egyptian strains have remained tightly grouped in recent years, staying fairly related while circulating similarly between chickens and humans in the data provided. In contrast, Turkey, Iraq, and Lebanon strains drifted away, showing different evolution paths. Older strains, such as ABQ58915.1 (Turkey 2005) and ABV24001.1 (Human Iraq 2006), exhibited greater divergence from the root, reflecting more accumulated mutations over time.

**Figure 7:**
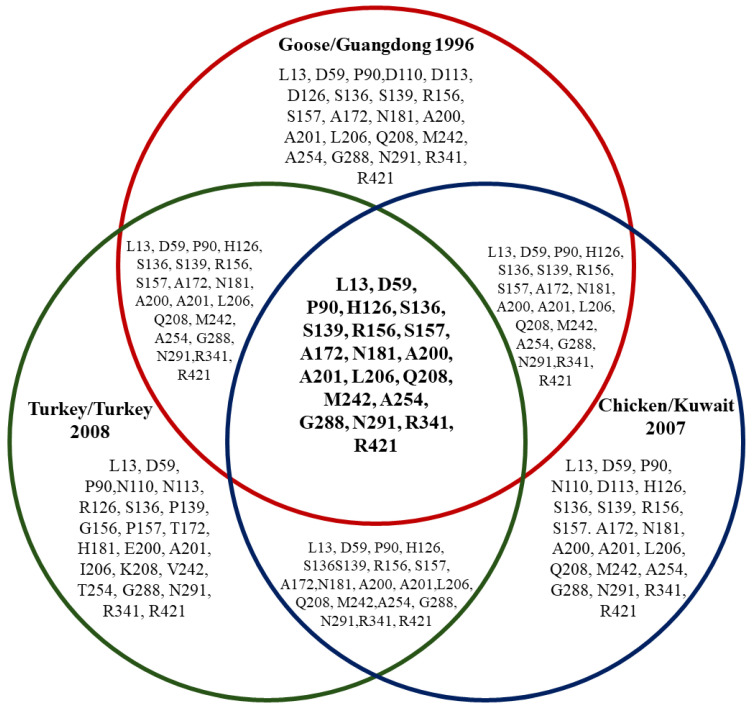
This Venn diagram emphasizes regional similarities in amino acids among three distinctive sequences: the original H5N1 Guangdong strain from 1996, the Kuwait strain from 2007, and the Turkey strain from 2008. In the figure, amino acid residues from each sequence are highlighted. All three sequences share 22 similar residues, shown in the middle intersection between all sequences.

**Figure 8:**
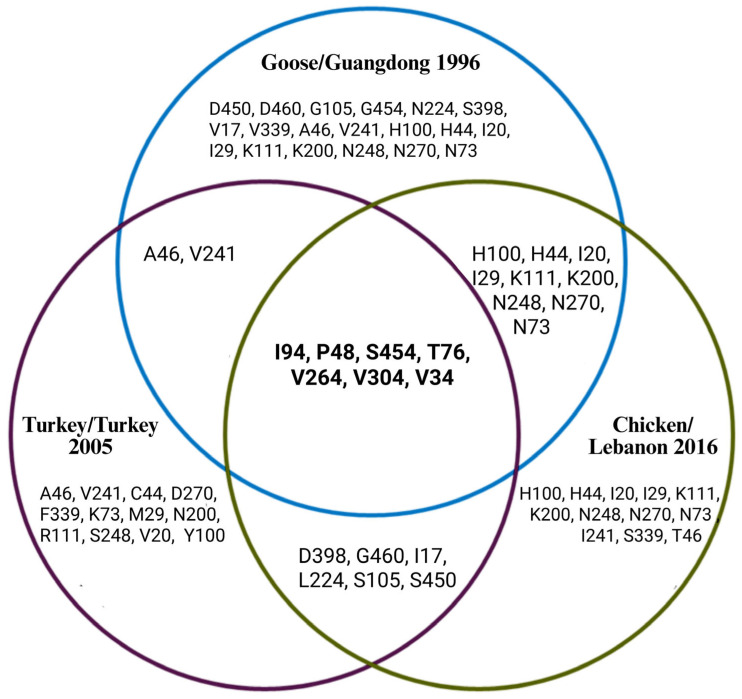
This Venn diagram shows the regional similarities in amino acids among three distinctive sequences: the original H5N1 Guangdong strain from 1996, the Turkey strain from 2005, and the Lebanon strain from 2016. In the figure, amino acid residues from each sequence are highlighted. All three sequences share 7 similar residues, which are shown in the middle intersection.

Furthermore, the amino acid analysis used key representative strains to compare HA and NA amino acid sequences separately. For HA, the original H5N1 China (YP_308669.1 Goose Guangdong 1996), Kuwait (ACA21577.1 Chicken Kuwait 2007), and Turkey (AEX88483.1 Turkey Turkey 2008) strains, respectively, were used. Thus, for NA, China 1996 (YP 308668.1 Goose Guangdong), Lebanon 2016 (AOE23190.1 Chicken), and Turkey 2005 (ABQ58915.1 Turkey) were used. After analyzing each dataset exclusively, the amino acid residues shown in the Venn diagrams in Figure 7 and Figure 8 were found. Several amino acid residues in HA from chosen strains were found to be conserved from the Middle East and the Guangdong reference strain, including L13, D59, P90, H126, S136, S139, R156, S157, A172, N181, A200, A201, L206, Q208, M242, A254, G288, N291, R341, and R421. The following residues were shared between the Goose/Guangdong 1996 and Turkey/Turkey 2008 strains: L13, D59, P90, H126, S136, S139, R156, S157, A172, N181, A200, A201, L206, Q208, M242, A254, G288, N291, R341, and R421. The following were shared between the Goose/Guangdong 1996 and Chicken/Kuwait 2007 strains: L13, D59, P90, H126, S136, S139, R156, S157, A172, N181, A200, A201, L206, Q208, M242, A254, G288, N291, R341, and R421. The following were shared between the Turkey/Turkey 2008 and Chicken/Kuwait 2007 strains: L13, D59, P90, H126, S136, S139, R156, S157, A172, N181, A200, A201, L206, Q208, M242, A254, G288, N291, R341, and R421. Additionally, unique residues were also detected for each strain: Turkey/Turkey 2008 (N113, R126, G156, P157, T172, H181, E200, I206, K208, V242, T254), Goose/Guangdong 1996 (D110, D113), and one for Chicken/Kuwait 2007 (H126) only. The amino acid sequences reveal that the Chicken/Kuwait 2007 strain is highly similar to Goose/Guangdong 1996, sharing many conserved residues without significant divergence. In addition, the NA from chosen strains shows amino acid site overlap and distinctions between the Goose/Guangdong 1996, Turkey/Turkey 2005, and Chicken/Lebanon 2016 H5N1 strains. Several positions such as I94, P48, S454, T76, V264, V304, and V34 are shared across all three groups, while each has a broader set of unique mutations: Goose/Guangdong 1996 (e.g., D450, D460, G105, G454, N224, S398, V17, V339); Turkey 2005 (e.g., C44, D270, F339, K73, M29); and Chicken/Lebanon 2016 (e.g., H100, H44, I20). Overall, the Venn diagrams’ overlapping regions show the strains’ similarities.

## 4. Discussion

Although H5N1 has been circulating in the Middle East since 2005, regional data remain limited and uneven, as highlighted by Hovmöller et al. (2010), where many studies rely on limited sample pools and single phylogenetic trees and may not capture the full complexity of H5N1 spread in different countries [40]. Egypt has contributed the majority of data sequenced, as seen in Figure 1, Figure 2, Figure 3, Figure 4, Figure 5 and Figure 6, while neighboring countries like Syria, Jordan, and Iran remain under-represented in public databases. This lack of balanced genomic surveillance has made it challenging to trace transmission routes, monitor viral evolution, or detect host-specific adaptation across the region [41,42]; without consistent, novel, and unrepetitive data from multiple countries, our understanding of how the virus spreads, evolves, and persists in different settings is incomplete, which results in the harder implementation of cross-border surveillance and control strategies.

The Middle East’s geographical and ecological diversity contributes to the emergence of various H5N1 clades, encompassing a range of habitats; for instance, environmental niche modeling has demonstrated that HPAI H5N1 presence in the Middle East is predicted in forests and mountainous regions, contrasting with patterns observed in other areas like West Africa [43]. Therefore, our study discovered several distinct clades circulating in the region, each with unique genetic signatures indicative of multiple introduction events and localized transmission chains. Our phylogenetic tree construction was parsimony-informative, revealing significant HA and NA protein relatedness among H5N1 strains through time, which shows that the clades branched off from multiple ancestral strains and were likely introduced through migratory birds, poultry trade, and other anthropogenic factors. Migratory bird flyways overlapping the region likely serve as a primary route, with birds carrying the virus from endemic areas in Asia and Africa [44]. As seen in Figure 1 and Figure 2, multiple sequences from Egypt, Israel, and other Middle Eastern countries cluster. The most noticeable point is that the Egyptian chicken isolates dominate the datasets, forming several tightly clustered subgroups with high bootstrap support; however, these high values confirm confidence in the topology, not necessarily low genetic divergence between the sequences. For example, in Figure 1, the bootstrap value of 100% between the KX644141.1 (Chicken Lebanon 2016) isolate and the NC_007362.1 (Goose Guangdong 1996) reference strain suggests a conserved lineage that may have persisted regionally with minimal mutation over time; this close association may imply that the Lebanese isolate retains genetic characteristics similar to the ancestral Goose Guangdong strain, possibly due to the persistence of specific viral lineages in the region. Due to some limitations in particular studies detailing the continuous circulation and evolution of H5N1 viruses in various regions, the World Health Organization (WHO) has reported the importance of monitoring genetic similarities and differences among strains to understand their evolution and spread [45]. Still, a study has documented the continuous evolution of H5N1 HPAI viruses across various poultry farming and production systems throughout Egypt, with clade 2.2.1.2 being the dominant cluster since 2011 [46], which further supports our findings of the observed clusters, reflecting continuous viral circulation and sustained endemicity within Egypt, which is likely driven by repeated local reintroductions or persistent lineage evolution within the poultry population.

Additionally, the analysis highlights Turkey and Lebanon’s roles in the regional transmission of H5N1. Figure 3 shows the HA amino acid genetic link between Turkey (AEX84883.1 Turkey 2008) and neighboring countries (ACA21577.1 Chicken Kuwait 2007). Both clusters are closely related to the reference sequence, suggesting cross-border viral transmission and potential host-switching events between poultry. Genetically similar strains from 2010 and 2011 were identified in chicken and turkey hosts from Egypt and Israel; sequences HM466695.1, JQ858472.1, and CY062609.1 from both countries aligned closely, suggesting that the virus likely crossed the Egyptian border to Israel and vice versa. This is further supported by the relationship between ADI58758.1 (Chicken Israel 2010) and CBW54817.1 (Chicken Egypt 2010), with a bootstrap value of 78%, and AFW18314.1 (Chicken Gaza 2008) clustering with AFW18183.1 (Chicken Menofia 2009), with a bootstrap value of 100%, pointing to regional spread or shared environmental conditions. The relatedness between these sequences likely reflects common transmission routes or ecological overlap. In contrast, strains like EU496389.1 and EU496390.1 (Egypt 2007) showed lower bootstrap support under 70%; this may be due to different reasons, like limited sequence data. Additionally, in Figure 5, the Lebanese isolate (AOE23190.1 Chicken 2016) stands out by clustering near the root with minimal divergence, which suggests early divergence compared to other regional strains; however, the tree highlights a critical point regarding human–animal transmission, where multiple human and chicken sequences from Egypt (2012–2016) are closely grouped, which may indicate connections and parallel evolution. Similarly, the nucleotide tree (Figure 1), particularly EU496390.1 Turkey Egypt 2007 and EU496389.1 Chicken Egypt 2007, suggests close genetic relatedness between poultry isolates from the same year and further emphasizes the virus’s zoonotic potential during that period, clearly highlighting the capability of the H5N1 strain to infect different hosts. Other studies have reported similar findings, where H5N1 strains from Turkey showed close genetic ties to isolates in neighboring countries, supporting patterns of reassortment among poultry populations [47]. Also, additional studies support Egypt’s role in sustained viral evolution and host adaptation by showing continuous viral circulation within its poultry [48]. Another closely related genetic relationship in Figure 1 was found between KR732525.1 Chicken Egypt 2006 and EF535821.1 Human Egypt 2007, which likely points to a direct transmission event between poultry and humans, which aligns with findings reported by Akpinar et al., who also observed similar regional spread dynamics [47]. Another instance was noted in 2015, where an isolated sample from a turkey in Israel (KT792922.1) was genetically correlated to a previously isolated H5N1 HA sample (KR063683.1) in 2014 in Egypt; the KR063683.1 sequence was isolated from a human host, raising concerns about the virus’s continued zoonotic potential and mortality risks. The molecular clock trees based on HA and NA protein sequences in Figure 4 and Figure 6 reveal a generally consistent and temporal divergence pattern. Older Egyptian isolates in Figure 4, like ACA29672.1 and ABY70828.1 (2007), showed greater evolutionary distance from the root node, reaching divergence values up to ~2.8793. These early strains clustered separately from later sequences, which supports the gradual diversification of viral lineages in Egypt over time; conversely, more recent isolates such as AQS24410.1, AQS24398.1, and AQS24406.1 (all from 2016) unexpectedly appeared closer to the root, with very small genetic distances (as low as 0.0131). This may suggest that these samples represent re-emerging or conserved lineages rather than newly evolved strains, possibly due to older variants’ persistence in the poultry population. Other sequences, such as AKC44576.1 (Human Egypt 2014) and ALJ35336.1 (Turkey Israel 2015), formed distinct distal branches, indicating independent introductions or localized evolution outside Egypt. Additionally, sequences from Lebanon, Turkey, and Israel were placed into their clades, showing moderate divergence and hinting at separate transmission routes. The tree reflects complex regional circulation patterns with divergence and occasional genetic conservation. As for Figure 6, the NA molecular clock tree also shows distinct temporal and geographic clustering patterns, where isolates from Egypt are often grouped closely, supporting cross-species transmission events within this region. In contrast, early isolates such as Turkey 2005 and Lebanon 2016 branched separately with greater genetic distances, with their early divergence pointing to independent viral introductions with limited subsequent spread or diversification in the region. These patterns collectively emphasize Egypt’s role as a major hotspot for H5N1 persistence and intraspecies transmission in the Middle East.

Additional analyses were conducted to gain further insights. The Venn diagram summary (Figure 4) revealed conserved residues identified across the Middle Eastern strains from the Goose/Guangdong 1996 strain, suggesting that these residues are critical for the function or stability of the hemagglutinin (HA) protein. Many of these conserved residues are located in functionally important regions, such as both the receptor-binding site (RBS) and fusion domains, and they are essential for viral host entry and are often under strong evolutionary constraints [49,50]. The high conservation of residues across strains (like L13, P90, S136, S139, R156, S157, A200, and M242) suggests strong purifying selection. The evolutionary pressure may have maintained these amino acids because of their functional importance, but any changes in these positions could have disrupted critical aspects of viral infectivity [51,52]. Immunologically, many of these conserved sites fall within known antigenic regions, which makes them promising candidates for broadly protective vaccine targets [52,53]. The shared amino acids between Goose/Guangdong 1996, Turkey/Turkey 2008, and Chicken/Kuwait 2007 indicate that the Turkey and Kuwait strains have evolved with minimal divergence and a close evolutionary lineage with the Guangdong reference strain. The high residue similarity between Turkey/Turkey 2008 and Chicken/Kuwait 2007 suggests close evolutionary ties; this was likely driven by regional transmission or shared environmental factors or is possibly linked to adaptation under different host immune pressures. Also, the Venn diagram in Figure 8 compares amino acid variations in the NA gene among Goose/Guangdong 1996, Turkey/Turkey 2005, and Chicken/Lebanon 2016 strains and revealed distinct and shared mutation patterns across regional lineages. The central overlaps highlight core mutations conserved among all groups, suggesting critical residues preserved during viral evolution to maintain essential NA function, where specific unique mutations reflect localized evolutionary pressures, likely linked to host adaptation or antiviral escape [54]. Interestingly, the Goose/Guangdong 1996 strain retained a broader set of original markers without major loss, consistent with its role as a reference ancestor [55]. The overlap patterns suggest that while core NA features remained conserved for functional integrity, additional mutations were acquired independently in various regional strains, promoting lineage separation and adaptation to different ecological or host environments, which aligns with previous findings that influenza NA genes exhibit both high conservation at functional sites and parallel diversification under host immune or antiviral pressure [8,56], which also supports our observation that specific unique mutations in regional strains reflect localized adaptation events and evolutionary responses to ecological and therapeutic pressures.

## 5. Surveillance, Policy, and Public Health Response to H5N1

Most human cases of H5N1 infection in Egypt were found to be caused by the human subclade 2.2.1, which was abundantly present in backyard poultry [57]. Genetic drift in the HA gene was initially observed when the virus first emerged, and it appeared at a profound level after 2008 when the virus was declared endemic [20]. The 2.2.1 and 2.2.1.1 subclades circulated among poultry populations between 2009 and 2011 [58]. The 2.2.1.1 subclade presumably arose due to continued vaccine pressure, diverging from the 2.2.1 subclade, which was already abundant in backyard poultry across the country [11]. Although 2.2.1.1 subclade detection in poultry declined after 2012, the emergence of the 2.2.1 subclade continued to prevail, evolving from new phylogenetic clusters [58]. The epidemiology of human H5N1 infection cases demonstrated that the case fatality rate was 34%, differing significantly between male and female patients, as higher fatality rates were found among females than males. Older age and slower hospitalization time contributed substantially to increasing fatality rates of H5N1 infections [20,59]. This highlights the importance of monitoring genetic drift and adapting vaccination strategies to address viral evolution effectively. Our findings—particularly in Egypt—emphasize the critical need for robust surveillance and biosecurity measures.

Egypt was considered the country with the highest reported number of cases worldwide, as it accounted for 37% of all H5N1 cases by 2015 [42], underscoring the region’s unique epidemiological dynamics. The persistence of the 2.2.1 subclade in Egypt is closely linked to inadequate biosecurity in backyard poultry farms and the high density of poultry farming, allowing continuous viral circulation throughout years and hosts. The data showing direct cross-species transmission between poultry and humans, as seen in the close genetic relationship between Chicken Egypt 2006 and Human Egypt 2007 (bootstrap 97%), highlight the zoonotic potential of the virus and the need for better biosecurity practices in preventing such events. Throughout the Middle East, many attempts to monitor and control the endemic spread of H5N1 virus strains have managed to confine their spread or, at the very least, decrease their infection and fatality rates; however, despite many attempts to monitor and control the virus (mainly through vaccination and surveillance), the dense populations and informal poultry farming practices in many Middle Eastern countries have made controlling the spread of H5N1 a significant challenge. Tourists and religious pilgrims, for example, who travel in large numbers to Middle Eastern countries, contribute to spreading diseases among the population [60]. Many studies have shown the effects of dense communities and diverse populations on the epidemiological spread of the virus since its emergence, pointing out the necessity of implementing surveillance and biosecurity strategies to control and prevent H5N1 spread effectively. However, it is crucial to point out that countries vary in their efficiency in planning and managing public health challenges and endemics according to the availability of resources and health management capabilities [61]. Various strategies should be implemented to establish strong preparedness against avian influenza. The implementation of surveillance systems across countries helps to ensure the vast detection of the H5N1 virus and track its spread and distribution through the conducting of phylogenetic analyses [62], thus elevating knowledge and understanding regarding viral transmission routes among a wide range of different hosts and species, highlighting species that directly contribute to animal–human transmission, therefore enabling the control of the spread of viral subtypes through the quarantining or culling of infected animals [63]. Risk assessment, management, and communication through efficient surveillance systems allow for early detection, enabling prompt intervention that prevents the emergence of potential outbreaks. Integrating advanced genomic technologies enhances biosecurity measures in predicting, combating, and lowering disease transmission risk. The one health approach addresses the challenges imposed by zoonotic diseases and provides effective protection measures for both animals and humans, so the interconnectedness of human, animal, and environmental health should be recognized by implementing the one health approach when assessing avian influenza [64]. International organizations, such as the World Organization for Animal Health (OIE), the Food and Agriculture Organization (FAO), and the World Health Organization (WHO), collaborate to share information, coordinate response efforts, and access resources for H5N1 surveillance and prevention [65]. Middle Eastern countries are in utmost need of supporting research and development areas to initiate an understanding of the ecology, transmission routes, and genetic diversity of H5N1, along with developing new diagnostic tools, which help in preparing for and preventing viral spread [66]. Research evolution helps address the gaps in our knowledge and generate peer-reviewed research papers that are scientifically supported to help devise new evidence-based methods for H5N1 surveillance and control. On the other hand, ignoring safety measures was observed to exacerbate viral infection in poultry farms, whereas applying them contributed significantly to mitigating the risk of infection [67]. Biosecurity in poultry farms is the primary defense against avian influenza spread. Many studies have explored various biosafety and biosecurity measures for preventing the transmission of H5N1. Many studies have shown that culling infected poultry effectively controls animal-to-animal and animal-to-human transmission by limiting direct contact and preventing infected animals from being consumed. In addition to the latter, restricting visitor access to farms and markets significantly helped reduce the risk of viral transmission to humans [68]. Disinfecting poultry farms, including cleaning farm tools and feeders, decreases the infection rate by 60% [69]. Various disinfectants, such as bleaching powder, lime, and potash, are used, with commercial farms spraying sheds and disinfecting feed sacks and litter equipment [70]. Furthermore, floor disinfection is also essential in maintaining a biologically safe environment and low transmission rates of avian influenza [71]. Poultry vaccination is highly valued as a biosecurity measure in combating the spread of the H5N1 virus [72]. However, it is noted that different animals react to the H5N1 vaccine differently. For instance, vaccinated chicken was observed to have elevated antibody titers after only a single dose. In contrast, ducks and geese produced a significantly lower antibody titer and needed at least two doses to attain protective immunity. These results highlight the need for implementing different vaccination programs for various species according to their vaccine response [73]. However, some species, such as ducks, were found to shed high titers of the virus while showing no symptoms, even though they were fully immunized [73], suggesting that tailored strategies are required for different poultry species.

Middle Eastern countries have faced significant challenges in combating the avian influenza virus through surveillance and biosecurity measures. Egypt, for example, has faced the most important challenge because it was the country most affected by the H5N1 strain, which led to the implementation of comprehensive vaccination campaigns, including poultry and human vaccinations. The government used locally produced and imported vaccines that were constantly updated to match the evolving H5N1 strain as part of a broader strategy that included surveillance systems, biosecurity measures, and public awareness campaigns [74]. Other Middle Eastern countries, such as Iraq, Syria, and Iran, have encountered several obstacles in achieving herd immunity to the avian influenza virus due to inadequate healthcare systems and insufficient resources for early disease detection and containment efforts, accompanied by recently occurring political issues [75,76,77,78]. For instance, Iraq is considered a bird migration route, thus establishing an easy pandemic point where highly controlled vaccination efforts are needed [79].

## 6. Study Limitations

While this study offers valuable insights, a few limitations that likely influenced the depth of the analysis should be recognized. One major challenge was sequence availability, which was significantly more complete than that of neuraminidase (NA) sequences in this region. This made it difficult to avoid bias during dataset selection and limited the ability to perform fully balanced comparative analyses. Additionally, the lack of full-length, high-quality NA data made it difficult to perform a fully parallel analysis; many NA entries in the NCBI database were highly repetitive, with several identical sequences, limiting their value for phylogenetic and evolutionary comparisons despite the apparent abundance. Another key limitation was the small dataset size, where only 62 HA and 37 NA sequences were included after filtering for quality and completeness. However, we worked to ensure diversity across hosts and geographic regions, though most sequences still came from Egypt, naturally skewing the dataset. All this uneven representation highlights the need for stronger, more consistent genomic surveillance across multiple countries, not just in places where the virus is heavily monitored.

## 7. Future Directions and Recommended Strategies

New influenza vaccinations are being developed to prepare for potential pandemic IAV outbreaks in the future. It is possible to identify novel viral targets for vaccine development using knowledge of the immune responses particular to influenza. Most of the vaccines used to date target viral surface hemagglutinin (HA), and they need to be updated as surface HA mutates quickly via reassortments or antigenic drifts [80]. A risk assessment of H5N1 vaccination is required to ensure the safety and efficacy of the vaccine. Prioritizing vaccine doses for highly susceptible individuals, for example, infants and older people, including those who suffer from chronic lung or heart diseases, metabolic deficiencies, or immunodeficiencies, is recommended [81]. Raising awareness and launching education campaigns exemplifies another key strategy in breaking the cycle of H5N1 transmission. It was found that communities with decent health education and knowledge were more likely to adopt preventive measures against zoonotic diseases and participate in practical collaborative applications to reduce their transmission [82]; therefore, educating the public, healthcare workers, and poultry workers about the risk of H5N1 transmission is highly recommended, in addition to the importance of practicing good hygiene and the proper handling of poultry products. Drug availability should be of the highest priority in countries where AIV outbreaks can occur. Suspected human H5N1 patients should receive immediate antiviral treatment, such as oseltamivir [83]. Besides treatment, quarantine is required to prevent further human–human transmission. Additionally, the potential impact of vaccine pressure on viral evolution should be critically evaluated. The divergence of the 2.2.1.1 subclade due to vaccine pressure and its subsequent decline after 2012 highlight the need for continuous monitoring and the adaptation of vaccination strategies. A reliance on vaccines without addressing broader biosecurity gaps, particularly in backyard poultry systems, may contribute to ongoing viral circulation and evolution. Vaccination strategies should be dynamic and periodically updated based on the latest phylogenetic analyses of circulating strains to better manage viral evolution, ensuring that vaccines target the most relevant viral subclades. Alongside vaccination, more robust biosecurity measures should be prioritized, which, combined with adaptive vaccination programs, will prevent the virus from circulating unchecked and reduce the risk of further evolution.

## 8. Conclusions

The phylogenetic analysis of H5N1 in the Middle East provides valuable insights into the virus’s diversity and how strains have evolved across time and locations. While our findings suggest regional patterns, the limited dataset and uneven country representation mean that some connections between clades, particularly across borders or hosts, should be interpreted with caution; however, the phylogenetic results supported our hypothesis, revealing clear host-specific and geographically influenced clade structures, with strong evidence of human–chicken transmission and regionally clustered evolution, particularly among Egyptian isolates. These findings underscore the ongoing viral evolution and adaptation of H5N1 in this region. Understanding the genetic relatedness of circulating strains can help guide targeted prevention efforts. Strengthening regional collaboration in surveillance, updating vaccination strategies based on current genetic data, and improving biosecurity measures in high-risk areas are critical steps in reducing future spread. Finally, addressing the data gaps from under-represented countries remains essential to building a more accurate and comprehensive picture of H5N1 dynamics in the Middle East.

## Data Availability

All the data are available in the NCBI Virus database.

## Supplementary Materials

The following supporting information can be downloaded at: https://www.mdpi.com/article/10.3390/v17050734/s1. Table S1: Comparative Analysis of Variable Amino Acid Sites in H5N1 Hemagglutinin Protein; Table S2: Comparative Analysis of Variable Amino Acid Sites in H5N1 Neuraminidase Protein; Table S3: HA and NA Amino Acid Composition.

## Abbreviations

AIV	Avian Influenza Virus
BLAST	Basic Local Alignment Search Tool
FAO	Food and Agriculture Organization
HA	Hemagglutinin
HPAI	Highly Pathogenic Avian Influenza
IAV	Influenza A Virus
LPAI	Low-Pathogenicity Avian Influenza
MEGA	Molecular Evolutionary Genetics Analysis
MENA	Middle East and North Africa
NA	Neuraminidase
NCBI	National Center for Biotechnology Information
NJ	Neighbor-Joining (phylogenetic method)
NNI	Nearest-Neighbor-Interchange
OIE	World Organization for Animal Health
Pi	Parsimony-Informative Sites
WHO	World Health Organization
